# The analyzation and clinical evaluation of ECLIA and CMIA in the detection of *Treponema pallidum*

**DOI:** 10.1097/MD.0000000000007139

**Published:** 2017-06-16

**Authors:** Jiansuo Zhou, Yongming Liang, Jie Zhang, Liyan Cui

**Affiliations:** Department of Clinical Laboratory, Peking University Third Hospital, Beijing, China.

**Keywords:** chemiluminescent magnetic microparticle immunoassay, electrochemiluminescence immunoassay analyzer, recombinant immunoblot assay, *Treponema pallidum*, *Treponema pallidum* particle agglutination test

## Abstract

To evaluate the clinical application value of electrochemiluminescence immunoassay analyzer (ECLIA) and chemiluminescent magnetic microparticle immunoassay (CMIA) in the detection of *Treponema pallidum* (TP).

A total of 1225 patients in Peking University Third Hospital was enrolled from June 2014 to October 2014. ECLIA and CMIA were applied to detect the serum anti-TP. The positive rate was analyzed. RIBA was taken as a golden standard to evaluate the sensitivity, the specificity, the positive predictive value, the negative predictive value, and the accuracy of ECLIA and CMIA. A correlation analysis between 2 assays was conducted, and that between assay and RIBA. We also evaluate the clinical value of TPPA in the detection of *T pallidum*.

The positive rate of CMIA and ECLIA is 10.63% and 9.89%, respectively, showing no statistically significant difference (*P* > .05). For CMIA, ECLIA, and TPPA, the sensitivity is 99.16%, 99.16%, and 99.16%, the specificity is 98.99%, 99.82%, and 100%, the positive predictive value is 91.47%, 98.33%, and 100%, the negative predictive value is 99.91%, 99.91%, and 99.91%, the coincidence rate is 99.01% (Kappa = 0.895), 99.75% (Kappa = 0.997), and 99.92% (Kappa = 0.998), respectively.

The result shows high correlation between ECLIA and CMIA. Both have high sensitivity and specificity and can be used as screening tests for the diagnosis of *T pallidum* in common condition.

## 1 Introduction

*Treponema pallidum* (TP) is the pathogenic factor for syphilis, which is a sexually transmitted disease (STD) and has the similar clinical signs and symptoms to other infectious diseases. TP infection can cause neurological, cardiovascular and other multisystem damage, clinical symptoms varied, longer course and the consequences are serious, even life-threatening.^[1]^ In recent years, the incidence of syphilis in China is rising, 80,406 cases were reported in 1999, the annual incidence rate was 6.50/100,000. 327,433 cases were reported in 2009, the annual incidence rate was 24.66/100,000, the annual incidence average increase 14.3%. 410,074 cases were reported in 2012, the annual incidence rate of 30.44/100,000 in 2009, the number of reported cases of syphilis in our class AB infectious disease report ranks third.^[2,3]^

In vitro culture technology of TP has not yet been resolved. Thus microbiological examination and serological screening has become the main way for the diagnosis of syphilis infection, especially serology. Early detection of disease is conducive to early diagnosis and prompt treatment.^[4]^ Since chemiluminescence immunoassay technology have high sensitivity, and strong specificity, but also has a fast, accurate, and automated, thus it has been widely used clinically. However, due to different reagents supplier, antigen detection using different segment, while also the sensitivity of detection reagents are different, so the sensitivity and specificity is difference from different reagent manufacturers and different methods.^[5–8]^ In this study, recombinant immunoblot assay (RIBA) as the golden standard reference method, clinical value of ROCHE E602 automatic electrochemiluminescence immunoassay analyzer (ECLIA) and ARCHITECT i2000SR automatic chemiluminescent magnetic microparticle immunoassay (CMIA) were studied. In addition, *Treponema pallidum* particle agglutination test (TPPA) is recognized the “gold standard” of syphilis antigen serology test by WHO, commonly used comparison of new method and reagent. We also assayed the sample by TPPA.

## 2 Materials and methods

### 2.1 Subjects

The study was approved by the local ethics committee. A total of 1225 patients in Peking University Third Hospital were enrolled from June 2014 to October 2014, they were divided into 4 groups. The first group is random serum samples, a total of 1000 cases, 438 of which were male, 562 female, aged 18 to 90 years old. The second group is serum of diagnosed definitely, including previously collected serum, a total of 100 cases, including 48 males and 52 females, aged 22 to 92 years old. For diagnosed definitely samples with a defined clinical stage, the staging was determined using the clinical and laboratory criteria. The third group was samples of other immune markers positive interference, which include hepatitis B virus (HBV), hepatitis C virus (HCV), Epstein–Barr virus (EBV), gestation, autoimmune disease, lymphoma, multiple myeloma, and other tumor, a total of 100 cases, including 49 males and 51 females, aged 18 to 92 years. The fourth group is CMIA threshold serum samples (0.9–5.0), a total of 25 cases, including 8 males and 17 females, aged 18 to 93 years old, signal/cut-off (S/CO) value of 1.02 to 4.87. The remaining serum after routine testing was collected for this experiment.

Syphilis is divided into stages; early syphilis consists of primary syphilis, secondary syphilis, and early latent syphilis, while late syphilis consists of late latent syphilis and tertiary syphilis. Our patients are divided into 5 stages, which include I, II, III, latent, and unknown stage syphilis.

### 2.2 Serum *T pallidum* specific antibodies determination

All the serum were separated by 3500 rpm centrifuge 10 minutes, after room temperature for 1 hour. The diagnosed serum, interference serum, and threshold serum were stored in −80°C, redissolve and mix well before testing.

All the serum were tested in ARCHITECT i2000SR (ARCHITECT Syphilis TP Abbott Laboratories, Wiesbaden, Germany, lot number 41537LI00) and ROCHE E601 (Elecsys Syphilis assay, Roche Diagnostics GmbH, Penzberg, Germany, lot number 17889301). All the results were expressed as a S/CO ratio, with a S/CO of <1.0 indicating a nonreactive result and a S/CO of ≥1.0 a reactive result. Serodia TPPA (Fujirebio, Tokyo, Japan) which are agglutination-based assays. For the Serodia TPPA the agglutination pattern was inspected and the results expressed as titers. The samples were then subjected to confirmatory testing with Immunoblot (Mikrogen Diagnostic, Martinsried, Germany) and it is a RIBA, which expressed the results as positive, negative, and indeterminate. All results were interpreted according to the manufacturers’ guidelines. Samples with initial nonreactive result are considered negative for *T pallidum* antibodies.

### 2.3 Statistics

All data were analyzed by statistical software SPSS 16.0. Kappa test was conducted to evaluate the consistency of qualitative results. Spearman correlation was used to show the correlation of quantitative results. Chi-square test was performed to analyze the qualitative data or categorical variable comparison. *P*-value <.05 was considered statistically significant. The positive rate, sensitivity, specificity, positive predictive value, negative predictive value and the coincidence rate were calculated using 2 × 2 contingency table about ECLIA, CMIA, and TPPA results.

## 3 Results

Of the 1225 enrolled patients, 12 samples (5 from random samples and 7 from borderline samples) were excluded from data analysis due to indeterminate RIBA results. Table 1 shows demographic and baseline clinical characteristics of the study patients. Among the total population, 119 had syphilis but 1094 not. Ten patients were diagnosed syphilis in the random serum samples and 9 patients in the borderline samples (Tables 1 and 2). Our results show that the positive rate of CMIA is 10.63% (129/1213), that of ECLIA is 9.89% (120/1213), that of TPPA is 9.63% (118/1213), the differences were not statistically significant (*P* > .05) (Table 3).

**Table 1:**
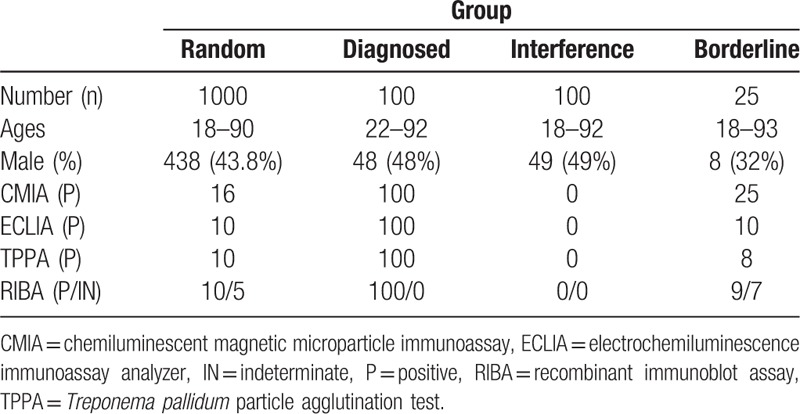
Clinical characteristics of the study group.

**Table 2:**
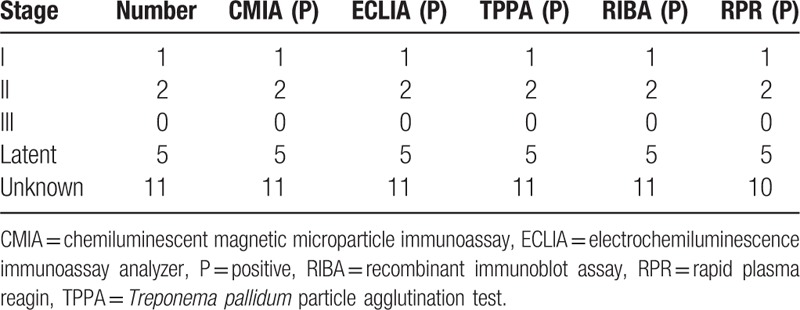
The result of confirmed diagnosis random and borderline serum in CMIA, ECLIA, TPPA, and RIBA.

**Table 3:**
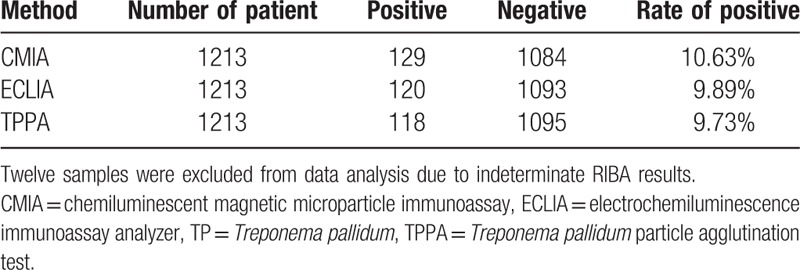
The result of TP in CMIA and ECLIA.

RIBA as the standard reference method, the diagnostic sensitivity, specificity, positive predictive value, and negative predictive value of CMIA is 99.16% (a/a + c), 98.99% (d/b + d), 91.47% (a/a + b), and 99.91% (d/c + d), respectively, and the coincidence rate of syphilis testing is 99.01 (a + d/a + b + c + d), Kappa = 0.895. That of ECLIA is 99.16% (a/a + c), 99.82% (d/b + d), 98.33% (a/a + b) and 99.91% (d/c + d), respectively, and the coincidence rate is 99.75% (a + d/a + b + c + d), Kappa = 0.997. That of TPPA is 99.16% (a/a + c), 100% (d/b + d), 100% (a/a + b), and 99.91% (d/c + d), respectively, and the coincidence rate is 99.92% (a + d/a + b + c + d), Kappa = 0.998 (Tables 4–7).

**Table 4:**
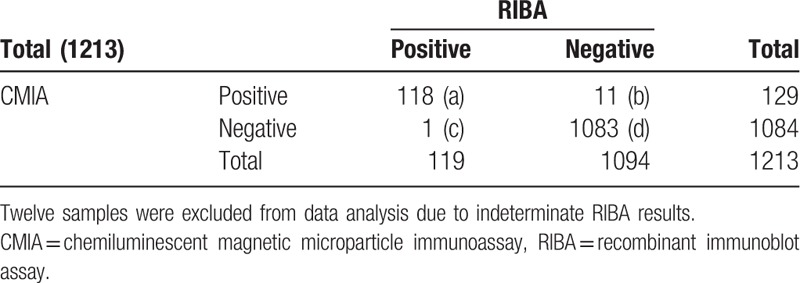
The comparison of result in CMIA and RIBA (n).

**Table 5:**
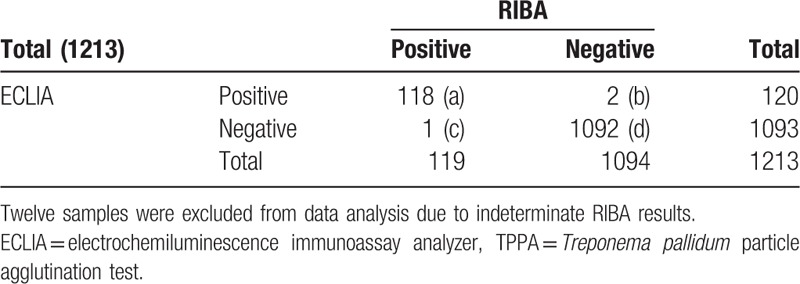
The comparison of result in ECLIA and RIBA (n).

**Table 6:**
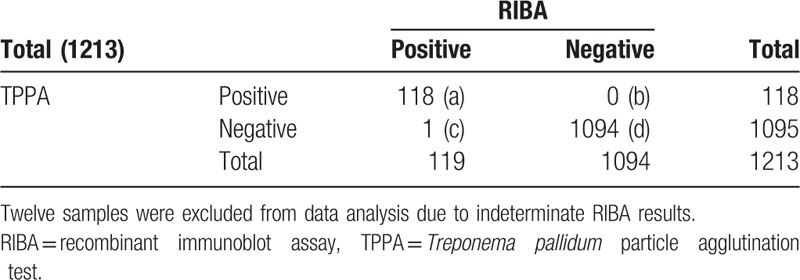
The comparison of result in TPPA and RIBA (n).

**Table 7:**
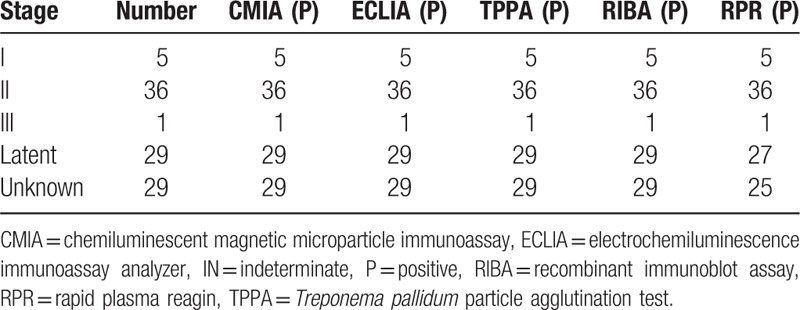
Sensitivity, specificity, PPV, NPV, and coincidence rate of all assays in the whole population.

**Figure 1:**
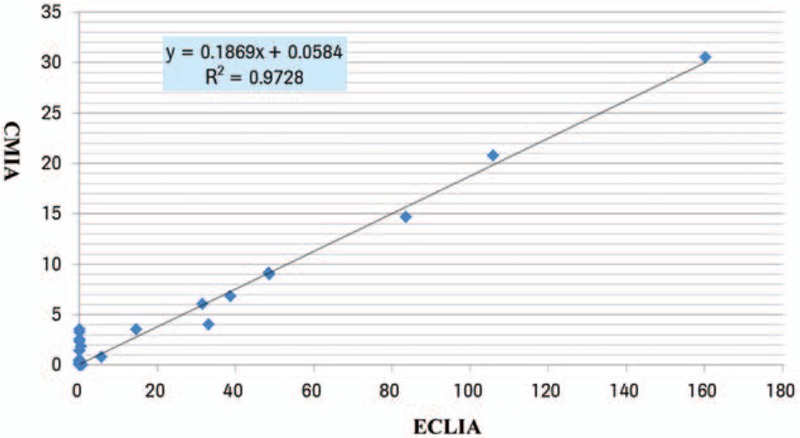
Comparison of the result of the CMIA and ECLIA in special groups.

The S/CO result of CMIA range from 0.02 to 30.5, that of ECLIA range from 0.065 to 160.4, the correlation coefficient R^2^ = 0.9728, which indicated that there was a good correlation between CMIA and ECLIA (Fig. 1).

In the 100 TP antibody positive serum of diagnosed definitely, CMIA and ECLIA both give 100% positive rate, indicated good consistency (Table 8). While the 100 positive interference serum, all of the CMIA, ECLIA, and TPPA give a 0% positive rate (Table 9).

**Table 8:**
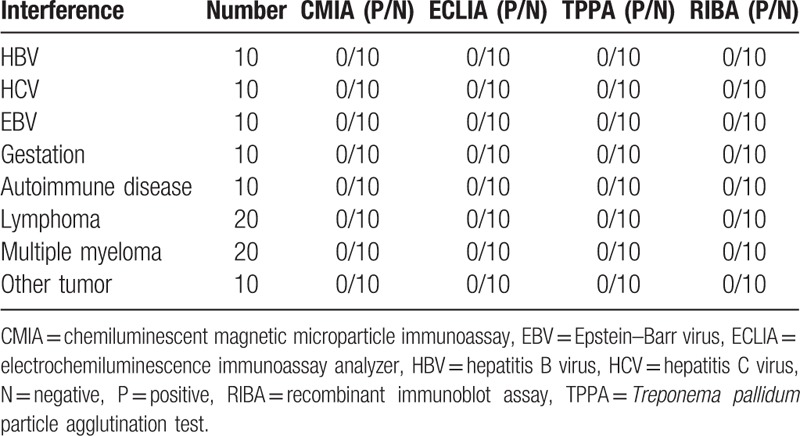
The result of confirmed diagnosis serum in CMIA, ECLIA, TPPA, RIBA, and RPR.

**Table 9:**
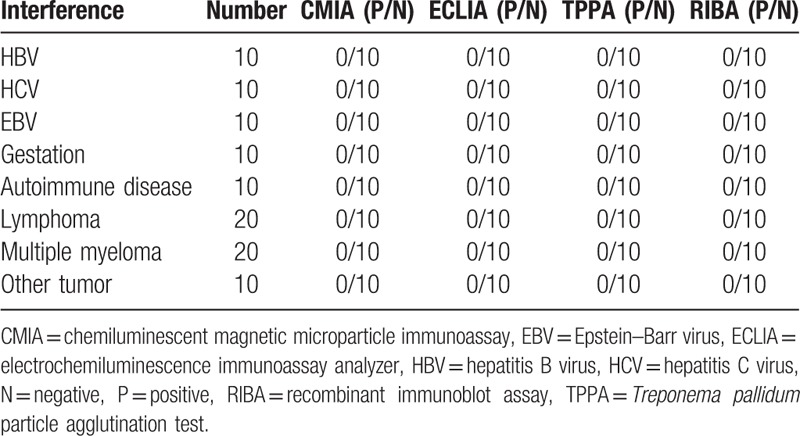
The result of interference serum in CMIA, ECLIA, and TPPA.

## 4 Discussion

Human is the main source of syphilis infection, it is divided into congenital syphilis and acquired syphilis by different infection way. The former is the pregnant woman infected with syphilis infect the fetus vertical transmission through the placenta. The later is transmitted through sexual contact after birth, or indirect means by blood transfusions, which consistent with the transmission of AIDS, syphilis infection can produce a variety of antibodies, mainly specific *T pallidum* antibodies and nonspecific antibodies, as long as the early detection and standard treatment can be cured. So it is very important for syphilis prevention and control to early detection of syphilis. Syphilis diagnosis mainly depends on laboratory tests and clinical symptoms, but it is difficult to make the diagnosis depend on clinical symptoms alone in the early onset, the laboratory plays an important role in the diagnosis of syphilis.

When human is infected with TP, chancre occur at the site of infection after about 3 weeks of incubation, then the syphilis antibodies can be detected in serum after 5 to 15 days. Serological tests play vital roles in the accurate diagnosis of syphilis and are divided into nontreponemal and treponemal tests. Treponemal tests are directed against *T pallidum* proteins with high specificity; these include fluorescent treponemal antibody-absorption (FTA-ABS), *Treponema pallidum* hemagglutination assay (TPHA), TPPA and enzyme immunoassay (EIA), chemiluminescence immunoassay (CMIA), syphilis IgM antibody test (TP-IgM), TP Western blot test (TP-WB). Among them FTA-ABS and RIBA are considered as confirmatory tests,^[9,10]^ but TPPA is the common used clinically because of high specificity and sensitivity. However, the TPPA have some inevitable limitations, such as complicated operation procedures, subjective results, time-consuming nature, and difficult automation, so it is not suitable for screening clinical samples. With the development of methodology, automated chemiluminescence immunoassay analyzer detection of syphilis antibodies gradually attention clinical examination.

In this study, we evaluated the capacity of CMIA and ECLIA in the determination of syphilis antibody, RIBA as a reference method. ECLIA gives a positive rate of 9.89%, CMIA gives 10.63%, while TPPA gives 9.63, the differences were not statistically significant (*P* > .05).

CMIA, ECLIA, and TPPA have good diagnosis sensitivity (99.16%, 99.16%, and 99.16%), specificity (98.99%, 99.82%, and 100%), positive predictive value (91.47%, 98.33%, and 100%), and negative predictive value (99.91%, 99.91%, and 99.91%), both them has good consistency with RIBA (97.96%, Kappa = 0.895; 99.67%, Kappa = 0.997), and the results of the CMIA and ECLIA methods have a good relevant with each other. Any of them can be used as a screening test for the diagnosis of syphilis under laboratory conditions.

In our study, CMIA and ECLIA give different results in 22 samples, 7 of which from random group, the other from borderline group. Regarding RIBA as the confirmatory test, CMIA give 11 false positive and 1 false negative, 2 false positive and 1 false negative occurs in ECLIA, 1 false negative occurs in TPPA. Such false-positive problem is attentioned gradually, the factors affect most biological false-positive results as follows: serum contain interference factors that lead to biological false positive result, such as rheumatoid factor, heterophilic antibodies, complement, some autoantibodies, cross-reactive substances.^[11]^ Malignant tumor or elderly patients with immune dysfunction may lead to false-positive easily.^[12]^ A kit containing recombinant antigen from recombinant gene expression may does not comply with the sequences.^[12]^ In our study, the specificity and sensitivity of CMIA and ECLIA are unable to reach 100%, but we can greatly reduce the number of false-positive samples by combine them. Therefore we recommend using one of the methods as the laboratory screening tests; the positive results were validated using another chemiluminescence immunoassay method to reduce the false-positive rate.

In summary, although CMIA and ECLIA exist some false-positive rate, but the false-negative rate is very low, and the chemiluminescence immunoassay method has the advantage of fast and high-throughput, in favor of a large number of specimens screening, any of them can be used as the preferred method of screening for syphilis serology diagnostic tests, but the false-positive rate cannot be ignored, so when the result of automatic chemiluminescence immunoassay analyzer for syphilis is positive, the confirmatory test is necessary, but due to RIBA is not often used in many laboratories and TPPA is common in most laboratories, we can use TPPA for the repeated measurement because of the low false-negative rate and zero false-positive rate. So we recommend use one of CMIA and ECLIA tests for screening and use of TPPA as confirmatory test due to low false-negative rate and zero false-positive rate and exclude RIBA test, as an alternative to manual TPPA test, and can be long-term application in clinical practice. A study of large sample size is needed to validate our results.
